# Heterogeneous hybrid signcryption for multi-message and multi-receiver

**DOI:** 10.1371/journal.pone.0184407

**Published:** 2017-09-08

**Authors:** Shufen Niu, Ling Niu, Xiyan Yang, Caifen Wang, Xiangdong Jia

**Affiliations:** College of Computer Science and Engineering, Northwest Normal University, Lanzhou, Gansu, China; Kaohsiung Medical University, TAIWAN

## Abstract

To achieve secure communication in heterogeneous cryptography systems, we present a heterogeneous hybrid signcryption scheme. The proposed scheme allows a sender in an identity-based cryptography system to send multi-message to multi-receiver in a certificateless cryptography system with different master keys. At the same time, all users are mapped to a distinct pseudo-identity for conditional identity privacy preservation. A trusted authority could trace the real identity when necessary. Compared with existing schemes, the proposed scheme is more practical for actual applications. In addition, the proposed scheme has indistinguishability against adaptive chosen ciphertext attacks and existential unforgeability against adaptive chosen message attacks under the random oracle model.

## Introduction

Diverse network systems have appeared with the development of technology. These systems utilize different cryptography techniques, such as public key infrastructure (PKI), identity-based cryptography (IBC), and certificateless cryptography (CLC). A cryptographic scheme should be constructed for secure communication in heterogeneous systems. Zheng [1] firstly proposed signcryption, a novel cryptographic primitive that functions as both digital signature and public key encryption in a single logical step that significantly costs lower than the traditional signature-then-encryption approach. Signcryption schemes are used to simultaneously achieve confidentiality, integrity, authentication, and non-repudiation for resource-constrained devices over low-bandwidth communication channels. Given those advantageous characteristics, heterogeneous signcryption is investigated. There are two types of heterogeneous signcryption between PKI and IBC: in type I, a sender in the PKI setting transmits a message to a receiver in the IBC setting; in type II, a sender in the IBC setting transmits a message to a receiver in the PKI setting. To achieve secure communication, Sun et al. [2] proposed type I schemes; these schemes, however, can only achieve outsider security. In 2011, Huang et al. [3] proposed a type II signcryption scheme with internal security. In 2013, Li et al. [4] proposed types I and II schemes that meet internal security requirements. Related heterogeneous signcryption paradigms have received considerable attention in recent years [5–8].

It is a practical way for large messages to use hybrid encryption perform secure communication. Hybrid encryption separates encryption into two parts: one part uses public key techniques to encrypt a one-time symmetric key, and the other part uses the symmetric key to encrypt the actual message. The public key encryption part of the algorithm is the key encapsulation mechanism (KEM), whereas the symmetric key encryption part is the data encapsulation mechanism (DEM). In 2003, a formal treatment of this paradigm originated in the work of Cramer and Shoup [9]. Dent [10, 11] studied the use of hybrid techniques to build signcryption schemes. He generalized KEM to signcryption KEM, which includes authentication. However, he only considered insider security for authenticity. In 2008, Tan [12] proposed full insider secure signcryption KEM in the standard model. Tan’s schemes are insider-secure for both authenticity and confidentiality. In 2005, Smart [13] provided an efficient key encapsulation for multiple parties. Sun et al. [14] proposed an IBC signcryption KEM for multiple recipients. Related hybrid signcryption or hybrid multiple receivers signcryption schemes can be found in [15–18].

Considering all the above literature, it is known that none of the existing multi-recipient heterogeneous hybrid signcryption schemes for IBC to CLC. However, in today’s complex network and application environment, the information security situation is also complicated and grim. The production and collection of the mass data results in information explosion lead the network communication become more complex and low effective due to diverse system [19, 20]and mathematical models [21, 22] of equipment environment. Then there need a scheme to achieve better communication between user with strong computing power and user who has weak computing power in heterogeneous system, the scheme also should handle large messages for sender to improve the efficiency of signcryption to multi-receiver.

Motivated by the above, considering with multi-PKG signcryption [23] and conditional privacy-preserving schemes [24], we propose a heterogeneous hybrid signcryption scheme for IBC to CLC which meets: (1) The private key generator (PKG) and key generation center (KGC) can produce different master keys and system parameters for different cryptography environments, which are more practical for heterogeneous systems. (2) The scheme is insider-secure for both authenticity and confidentiality, and the formal definitions and security models for heterogeneous hybrid signcryption scheme are also given. (3) Each user maps a distinct pseudo-identity to achieve conditional identity privacy preservation. A trusted authority could trace the real identity when necessary. (4) Use hybrid signcryption to implement a sender signcrypt multi-message to multi-receiver in once signcryption.

The rest of this paper is organized as follows: preliminary information is given in section 2. The framework and security model are presented in section 3. The heterogeneous hybrid signcryption for multi-message and multi-receiver (MHHSC) scheme is proposed in section 4. The security proof is presented in section 5. The performance evaluation of the proposed scheme is discussed in section 6. Finally, the conclusion is provided in section 7.

## Preliminary

In this section, we describe bilinear maps and hard problems. Let consider two cyclic groups *G*_1_ and *G*_2_ with the same prime order *q*, and let *P* is a generator of *G*_1_. A bilinear map *e*: *G*_1_ × *G*_1_ → *G*_2_ need satisfy the following properties:

1. Bilinearity: For all *P*, *Q*, *R* ∈ *G*_1_, and $a,b\in Z_{q}^{*}$, *e*(*P* + *R*, *Q*) = *e*(*P*, *Q*)*e*(*R*, *Q*). Also *e*(*aP*, *bQ*) = *e*(*P*, *Q*)^*ab*^.
2. Non-degeneracy: There exists *P*, *Q* ∈ *G*_1_, such that *e*(*P*, *Q*)≠1.
3. Computability: *e*(*P*, *Q*) can be computed for *P*, *Q* ∈ *G*_1_.

*Definition 1. Given two groups* *G*_1_ *and* *G*_2_ *of the same prime order* *q*, *a bilinear map* *e*: *G*_1_ × *G*_1_ → *G*_2_, *and a generator* *P* *of* *G*_1_, *the decisional bilinear Diffie-Hellman(DBDH) problem is to decide whether* *T* = *e*(*P*, *P*)^*abc*^ *for given* (*P*, *aP*, *bP*, *cP*) *and* *T* ∈ *G*_2_.

*Definition 2. Variants decisional bilinear Diffie-Hellman(VDBDH) problem is to decide whether* *T* = *e*(*P*, *P*)^*abc*^−1^^ *for given* (*P*, *aP*, *bP*, *cP*, *c*^−1^ *P*) *and* *T* ∈ *G*_2_.

*Definition 3. Variants computational bilinear Diffie-Hellman(VCBDH) problem is to compute* *T* = *e*(*P*, *P*)^*abd*^−1^^ *for given* (*P*, *aP*, *bP*, *dP*, *d*^−1^ *P*).

## Framework and security model for MHHSC

### MHHSC KEM

MHHSC KEM consists of five algorithms:

- Setup: With a security parameter *ℓ* as the input, the PKG and KGC generate their own master key and output the system parameters *params*.
- Anony-IBC-KG: The algorithm runs by the PKG of the IBC system. With a user’s real identity *RID*_*A*_ and *ID*_*A*,1_ as the input, the algorithm generates the corresponding private key *sk*_*A*_ and pseudo-identity *ID*_*A*_.
- Anony-CLC-KG: The algorithm runs by the KGC of the CLC system. With a user’s real identity *RID*_*B*_*i*__ and *ID*_*B*_*i*_,1_ as the input, the algorithm generates the corresponding partial private key *D*_*B*_*i*__, secret key *sk*_*B*_*i*__, public key *pk*_*B*_*i*__ and pseudo-identity *ID*_*B*_*i*__.
- Encap: Give the sender’s identity (*Q*_*A*_, *ID*_*A*_), and private key *sk*_*A*_, receiver identity (*pk*_*B*_*i*__, *ID*_*B*_*i*__, *Q*_*B*_*i*__(*i* = 1, 2, ⋯, *n*)), the algorithm outputs the encapsulation key *K* and encapsulation *φ*.
- Decap: Give the sender’s identity (*Q*_*A*_, *ID*_*A*_), receiver secret key, and public key (*D*_*B*_*i*__, *sk*_*B*_*i*__), (*pk*_*B*_*i*__, *ID*_*B*_*i*__), the algorithm outputs the encapsulation key *K* or the symbol ⊥.

### DEM

DEM is a symmetric encryption scheme that requires security for confidentiality and unforgeability. DEM consists of the following two algorithms:

- Enc: Take message *M* and encapsulation key *K* as input, the ciphertext *C* is then output. We denote this as *C* ← *DEM*.*Enc*(*K*, *M*).
- Dec: Take a key *K* and the ciphertext *C* as input, the message *M* or error symbol ⊥ is output.

### MHHSC HSC

The proposed MHHSC scheme consists of MHHSC KEM and DEM as follows:

- Setup, Anony-IBC-KG, and Anony-CLC-KG: Same as 3.1 MHHSC KEM.
- Signcrypt: Use Encap in 3.1 MHHSC KEM to obtain (*K*, *ϕ*), use Enc in 3.2 DEM to obtain a ciphertext *C*, output *σ* ← (*C*, *ϕ*).
- Unsigncrypt: Use Decap in 3.1 MHHSC KEM to obtain *K*, use Dec in 3.2 DEM to obtain message *M*, then check the equation. If it holds, receive *M*. Otherwise, output the symbol ⊥.

### Security notions

In the proposed scheme, the confidentiality property is defined based on the concept of indistinguishability against adaptive chosen ciphertext attacks (IND-CCA2), which considers two types of adversaries with different capabilities. A type I adversary acts as a dishonest user, whereas a type II adversary acts as a malicious KGC that can obtain the master secret key of KGC. The authenticity property is defined basis on existential unforgeability against adaptive chosen message attacks (EUF-CMA).

*Definition 4. (Confidentiality) A heterogeneous hybrid signcryption scheme is said achieved IND-CCA2, if no probabilistic polynomial time adversary* *A*_1_ *has a non-negligible advantage in the following game:*

Setup: The challenger *C* runs the Setup algorithm and sends system parameters and public keys to *A*_1_, whereas the KGC’s master key is kept secret. $ID_{B_{i}}^{*}\left(i=1,2,\cdots ,n\right)$ is the target identity.

Phase 1. *A*_1_ can ask several kinds of queries to the following random oracles:

- Partial private key query: Submit a query on *ID*_*B*_*j*__. If $ID_{B_{j}}\neq ID_{B_{i}}^{*}$ (*i* = 1, 2, ⋯, *n*), then return *D*_*B*_*j*__. Otherwise, *C* aborts.
- Unsigncrypt query: Submit an unsigncrypt query under *ID*_*A*_, *ID*_*B*_*j*__ and ciphertext *σ*. If $ID_{B_{j}}\neq ID_{B_{i}}^{*}\left(i=1,2,\cdots ,n\right)$, then *C* runs the formal unsigncrypt algorithm and returns the answer. Otherwise, *C* searches the list and computes *M*. Then, check the equation. If holds, *M* is returned. Otherwise, ⊥ is output.

Challenge: *C* decides when the Phase 1 ends. *A*_1_ selects two plaintexts *M*_0_, *M*_1_ of the same length, and *ID*_*A*_, *ID*_*B*_*j*__(*j* = 1, 2, ⋯, *n*) to *C*, which wants to challenge. If $ID_{B_{j}}\neq ID_{B_{i}}^{*}\left(i=1,2,\cdots ,n\right)$, *C* fails and aborts. *A*_1_ is not allowed to ask the partial private key of $ID_{B_{i}}^{*}$. Then, *C* selects *b* ∈ {0, 1} and runs the corresponding algorithms to obtain the ciphertext σ* transmits to *A*_1_.

Phase 2. *A*_1_ can perform queries as in Phase 1. *A*_1_ cannot query the key extraction for the target identities and should not query the unsigncrypt of σ*.

Guess: Finally, *A*_1_ produces a bit *b*′, *A*_1_ wins the game if *b*′ = *b*.

*Definition 5. (Confidentiality) A heterogeneous hybrid signcryption scheme is said achieved IND-CCA2, if no probabilistic polynomial time adversary* *A*_2_ *has a non-negligible advantage in the following game:*

Setup: The challenger *C* runs the Setup algorithm that sends system parameters and public keys to *A*_2_. $ID_{B_{i}}^{*}\left(i=1,2,\cdots ,n\right)$ is the target identity.

Phase 1. *A*_2_ can ask several queries to the following random oracles:

- Public key query: Submit a public key query on *ID*_*B*_*j*__. If $ID_{B_{j}}=ID_{B_{i}}^{*}$ (*i* = 1, 2, ⋯, *n*), update *PK*-list with (*ID*_*B*_*j*__, ⊥, *cP*), and return *pk*_*B*_*j*__.
- Unsigncrypt query: Submit an unsigncrypt query under *ID*_*A*_, *ID*_*B*_*j*__ and ciphertext *σ*. If $ID_{B_{j}}\neq ID_{B_{i}}^{*}\left(i=1,2,\cdots ,n\right)$, *C* runs the formal unsigncrypt algorithm and returns the answer. Otherwise, *C* searches the list and computes *M*. Then, check the equation. If the equation holds, return *M*. Otherwise, ⊥ is output.

Challenge: *C* decides when Phase 1 ends. *A*_2_ selects two plaintexts *m*_0_, *m*_1_ of the same length, and *ID*_*A*_, *ID*_*B*_*j*__(*j* = 1, 2, ⋯, *n*) to *C*, which wants to challenge. If $ID_{B_{j}}\neq ID_{B_{i}}^{*}\left(i=1,2,\cdots ,n\right)$, *C* fails and aborts. *A*_2_ is not allowed to query for the secret key of $ID_{B_{i}}^{*}$. Then *C* selects *b* ∈ {0, 1} and runs the corresponding algorithms to obtain the ciphertext σ* transmits to *A*_2_.

Phase 2. *A*_2_ can perform queries as Phase 1. *A*_2_ cannot query the key extraction for the target identities and should not query the unsigncrypt of σ*.

Guess: Finally, *A*_2_ produces a bit *b*′, and *A*_2_ wins the game if *b*′ = *b*.

*Definition 6. (Unforgeability) A heterogeneous hybrid signcryption scheme is said achieved EUF-CMA, if no probabilistic polynomial time forger* *F* *has a non-negligible advantage in the following game:*

Setup: The challenger *C* runs the Setup algorithm and sends system parameters and public keys to *F*, whereas the PKG’s master key is kept secret. $ID_{A}^{*}$ is the target identity.

Attack: *F* issues several kinds of queries.

- Private key query: Submit a query on *ID*_*A*_. If $ID_{A}\neq ID_{A}^{*}$. Then return *sk*_*A*_. Otherwise, *C* aborts.
- Signcrypt query: Submit a signcrypt query under *ID*_*A*_, ${\left\{ID_{B_{i}}\right\}}_{i=1}^{n}$. If $ID_{A}\neq ID_{A}^{*}$, runs the formal signcrypt algorithm and returns ciphertext *σ*. Otherwise, *C* computes *σ* to satisfy the equation and returns *σ* to *F*.

Forgery: Finally, *F* outputs σ* under $ID_{A}^{*}$, $ID_{A}^{*}$ cannot query the private key, *F* wins if Unsigncrypt does not return ⊥.

## Heterogeneous hybrid signcryption for multi-message and multi-receiver

The MHHSC scheme will be discussed in this section. The proposed scheme involves four parties: PKG, KGC, sender *ID*_*A*_, and *n* receivers ${\left\{ID_{B_{i}}\right\}}_{i=1}^{n}$, allowing *ID*_*A*_ to send *m* messages to *n* receivers ${\left\{ID_{B_{i}}\right\}}_{i=1}^{n}$. *KDF* in scheme denotes a key extract function in *G*_1_. Moreover, PKG and KGC can calculate pseudo-identities for users in their system, key pairs or partial private keys of all users are generated by PKG or KGC via the pseudo-identities.

- Setup: Let *G*_1_ and *G*_2_ be two cyclic groups with prime order *q*, where *G*_1_ is additive and *G*_2_ is multiplicative, and *P* is the generator of *G*_1_. Let *e*: *G*_1_ × *G*_1_ → *G*_2_ be an admissible bilinear map, a key extract function *KDF*: ${\left\{0,1\right\}}^{l_{m}}\to G_{1}$ (*l*_*m*_ is the length of a key).
  1. PKG randomly selects $s_{1}\in Z_{q}^{*}$ and two hash functions: *H*_0_: *G*_1_ → {0, 1}*, *H*_1_: {0, 1}* → *G*_1_ computes *P*_1_ = *s*_1_ *P*, where *s*_1_ is a master secret key that only the PKG knows.
  2. KGC randomly selects $s_{2}\in Z_{q}^{*}$ and four hash functions: *H*_2_: *G*_1_ → {0, 1}*, *H*_3_: {0, 1}* → *G*_1_, $H_{4}:G_{2}\to Z_{q}^{*}$, $H_{5}:{\left\{0,1\right\}}^{*}\to Z_{q}^{*}$ calculates *P*_2_ = *s*_2_ *P*, where *s*_2_ is a master secret key that only the KGC known.
  Public *params* = <*e*, *P*, *P*_1_, *P*_2_, *G*_1_, *G*_2_, *H*_0_, *H*_1_, *H*_2_, *H*_3_, *H*_4_, *H*_5_, *KDF*> and keep *s*_1_, *s*_2_ secret respectively.
- Anony-IBC-KG: Users in IBC obtain their private key as follows:
  1. Sender *A* randomly selects $k_{A}\in Z_{q}^{*}$ calculates *ID*_*A*,1_ = *k*_*A*_ *P* and transmits (*RID*_*A*_, *ID*_*A*,1_) to PKG, where *RID*_*A*_ is the real identity of sender *A*. PKG calculates *ID*_*A*,2_ = *RID*_*A*_ ⊕ *H*_0_(*s*_1_ *ID*_*A*,1_, *T*), where *T* denotes the valid period of this pseudo-identity. Finally, the identity os sender *A* is *ID*_*A*_ = (*ID*_*A*,1_, *ID*_*A*,2_, *T*).
  2. PKG generates a private key for IBC users as $sk_{A}=s_{1}^{-1}Q_{A}$, where *Q*_*A*_ = *H*_1_(*ID*_*A*_). (*sk*_*A*_, *Q*_*A*_, *ID*_*A*_) is sent to *A* via a secure path.
- Anony-CLC-KG: Users in CLC obtain their partial private key as follows:
  1. Receiver *B*_*i*_(*i* ∈ {1, 2, ⋯, *n*}) randomly selects $k_{B_{i}}\in Z_{q}^{*}$ calculates *ID*_*B*_*i*_,1_ = *k*_*B*_*i*__ *P* and transmits (*RID*_*B*_*i*__, *ID*_*B*_*i*_,1_) to KGC, where *RID*_*B*_*i*__ is the real identity of receiver *B*_*i*_. KGC calculates *ID*_*B*_*i*_,2_ = *RID*_*B*_*i*__ ⊕ *H*_2_(*s*_2_ *ID*_*B*_*i*_,1_, *T*_*i*_), where *T*_*i*_ denotes the valid period of this pseudo-identity. Finally, the identity of receiver *B*_*i*_ is *ID*_*B*_*i*__ = (*ID*_*B*_*i*_,1_, *ID*_*B*_*i*_,2_, *T*_*i*_).
  2. KGC generates the partial private key for CLC users as $D_{B_{i}}=s_{2}^{-1}Q_{B_{i}}$, where *Q*_*B*_*i*__ = *H*_3_(*ID*_*B*_*i*__). (*D*_*B*_*i*__, *Q*_*B*_*i*__, *ID*_*B*_*i*__) is sent to *B*_*i*_ via a secure path.
  3. *B*_*i*_ randomly selects the secret value $x_{B_{i}}\in Z_{q}^{*}$ to compute *sk*_*B*_*i*__ = *x*_*B*_*i*__ *D*_*B*_*i*__, *pk*_*B*_*i*__ = *x*_*B*_*i*__ *P*.
- Signcrypt: A sender *A* signcrypts *n* messages *m*_*i*_(*i* = 1, 2, ⋯, *n*) to *n* receiver *B*_*i*_(*i* = 1, 2, ⋯, *n*) as follows:
  1. Randomly selects $r_{1},r_{2}\in Z_{q}^{*}$, and computes *U*_1_ = *r*_1_ *P*_2_, *U*_2_ = *r*_1_*Q*_*A*_.
  2. Compute $V_{i}=e{\left(P,Q_{B_{i}}\right)}^{r_{1}}$, $R_{i}=e{\left(pk_{B_{i}},Q_{B_{i}}\right)}^{r_{1}}$, *φ*_*i*_ = *r*_2_ ⊕ *H*_4_(*V*_*i*_) and let *φ* = (*φ*_1_, *φ*_2_, ⋯, *φ*_*n*_).
  3. Compute *C* = *DEM*.*Enc*(*K*, *M*) where *K* = *KDF*(*r*_2_) and *M* = (*m*_1_ ⊕ *R*_1_‖*m*_2_ ⊕ *R*_2_‖⋯‖*m*_*n*_ ⊕ *R*_*n*_).
  4. Compute *h*_*i*_ = *H*_5_(*U*_1_, *U*_2_, *M*, *R*_*i*_, *V*_*i*_, *ID*_*A*_, *ID*_*B*_*i*__).
  5. Compute *S*_*i*_ = (*r*_1_ + *h*_*i*_)*sk*_*A*_ and let *S* = (*S*_1_, *S*_2_, ⋯, *S*_*n*_).
  Return ciphertext *σ* = (*C*, *ϕ* ← (*U*_1_, *U*_2_, *S*, *φ*)).
- Unsigncrypt: After receiving a ciphertext *σ* = (*C*, *ϕ* ← (*U*_1_, *U*_2_, *S*, *φ*)), the receiver *B*_*i*_(*i* ∈ {1, 2, ⋯, *n*}) decrypts *σ* as follows:
  1. Compute *V*_*i*_ = *e*(*U*_1_, *D*_*B*_*i*__), *R*_*i*_ = *e*(*U*_1_, *sk*_*B*_*i*__) and obtain *r*_2_ = *φ*_*i*_ ⊕ *H*_4_(*V*_*i*_).
  2. Recover *M* = *DEM*.*Dec*(*K*, *C*) where *K* = *KDF*(*r*_2_). Receiver *B*_*i*_ recovers own message *m*_*i*_ = (*m*_*i*_ ⊕ *R*_*i*_) ⊕ *R*_*i*_.
  3. Compute *h*_*i*_ = *H*_5_(*U*_1_, *U*_2_, *M*, *R*_*i*_, *V*_*i*_, *ID*_*A*_, *ID*_*B*_*i*__).
  4. Accept the message if and only if the following equation holds:
    $$\begin{array}{c} e\left(P_{1},S_{i}\right)=e\left(P,U_{2}+h_{i}Q_{A}\right). \end{array}$$

Note that conditional privacy preservation for each user is mapped to a distinct pseudo-identity *ID*_*U*_ = (*ID*_*U*,1_, *ID*_*U*,2_, *T*). PKG or KGC can retrieve the real identity from any pseudo-identity by *RID*_*U*_ = *ID*_*U*,2_ ⊕ *H*_*i*_(*sID*_*U*,1_, *T*) for any disputed event. In addition, the pseudo-identity *ID*_*U*_ is generated by both users and PKG or KGC. Hence, only the PKG or KGC that knows the master secret *s* can retrieve the real identity *RID*_*U*_ from *ID*_*U*_.

## Security proof

In this section, we prove that the proposed IBC to CLC hybrid scheme achieves the security requirements of confidentiality and unforgeability. To demonstrate the security of our scheme, we assume that the adversary asks *q*_*H*_*i*__ queries to *H*_*i*_ for *i* = 1, 2, 3, 4, 5, *q*_*u*_ queries to unsigncryption; *q*_*s*_ queries to the signcryption; *q*_*ppk*_ queries to the partial private key; *q*_*sk*_ queries to the secret key; *q*_*pk*_ queries to the public key extraction; and *q*_*pkr*_ queries to the public key replacement.

### Confidentiality

Theorem 1. The above MHHSC scheme is secure against adaptive chosen ciphertext attacks in the standard model assuming that the VDBDH and DBDH problems are difficult.

This theorem follows lemmas 1 and 2. Lemma 1 reveals that adversary *A*_1_ can not distinguish *M*. Lemma 2 proves that although adversary *A*_2_ can obtain *M*, it cannot distinguish message *m*_*j*_ for *ID*_*B*_*j*__.

*Lemma 1. In the random oracle, if there is an IND-CCA2 adversary* *A*_1_ *has an advantage* *ϵ* *against MHHSC, then there is an algorithm* *C* *that solves the VDBDH problem with an advantage* $\frac{\epsilon -\left(q_{u}-q_{H_{4}}q_{s}-q_{s}^{2}\right)/2^{k-1}}{2q_{H_{3}}}$.

Proof. We construct a simulator *C* that use *A*_1_ to decide whether *T* = *e*(*P*, *P*)^*abc*^−1^^ by providing a random instance (*P*, *aP*, *bP*, *cP*, *c*^−1^ *P*, *T*) as the VDBDH problem. This proof consider the indistinguishability of *M*.

Setup: At the beginning, *C* sets *P*_2_ = *cP* and proves the system parameters to the attacker *A*_1_. The target identity is $ID_{B_{i}}^{*}\left(i=1,2,\cdots ,n\right)$.

Phase 1. *A*_1_ requests a number of queries. *C* keeps the *H*_*i*_-list (*i* = 1, 2, 3, 4, 5) and *PK*-list which are used to record answers to the corresponding *H*_*i*_ query and public key query.

- *H*_3_ query: Input an identity *ID*_*B*_*j*__. If $ID_{B_{j}}\neq ID_{B_{i}}^{*}\left(i=1,2,\cdots ,n\right)$, randomly select $t_{j}\in Z_{q}^{*}$, calculate *Q*_*B*_*j*__ = *t*_*j*_ *P*. Otherwise, calculate *Q*_*B*_*j*__ = *bP* place (*ID*_*B*_*j*__, *t*_*j*_, *Q*_*B*_*j*__) in the *H*_3_-list, and return *Q*_*B*_*j*__.
- *H*_4_ query: If (*V*_*j*_, *h*_4_) exists in the *H*_4_-list, return *h*_4_. Otherwise, check if VDBDH oracle returns 1 when queried with the tuple (*aP*, *bP*, *c*^−1^ *P*, *V*_*j*_). If this is the case, *C* returns *V*_*j*_ = *e*(*P*, *P*)^*abc*^−1^^ and stops. Otherwise, randomly select $h_{4}\in Z_{q}^{*}$ update the *H*_4_-list, and return *h*_4_.
- *H*_*i*_(*i* = 0, 1, 2, 5) query: Upon receiving an *H*_*i*_ query, if the corresponding query exists in the *H*_*i*_-list, return it to *A*_1_. Otherwise, *C* randomly selects an integer as the query result and returns it to *A*_1_. Meanwhile, *C* places the query result into the *H*_*i*_-list.
- Partial private key query: Upon receiving a partial private key query on *ID*_*B*_*j*__. If $ID_{B_{j}}\neq ID_{B_{i}}^{*}\left(i=1,2,\cdots ,n\right)$, retrieves the corresponding (*ID*_*B*_*j*__, *t*_*j*_, *Q*_*B*_*j*__) from the *H*_3_-list and sets *D*_*B*_*j*__ = *t*_*j*_*c*^−1^ *P* return *D*_*B*_*j*__. Otherwise, *C* aborts.
- Public key query: When *C* receives a public key query on *ID*_*B*_*j*__, if there exists (*ID*_*B*_*j*__, *x*_*B*_*j*__, *pk*_*B*_*j*__) in the *PK*-list, then *C* returns *pk*_*B*_*j*__; otherwise, *C* randomly selects $x_{B_{j}}\in Z_{q}^{*}$, computes *pk*_*B*_*j*__ = *x*_*B*_*j*__ *P*, places (*ID*_*B*_*j*__, *x*_*B*_*j*__, *pk*_*B*_*j*__) into the *PK*-list, and returns *pk*_*B*_*j*__ as the answer.
- Replace public key: When *C* receives a replace public key query on *ID*_*B*_*j*__, *C* first finds (*ID*_*B*_*j*__, *x*_*B*_*j*__, *pk*_*B*_*j*__) on the *PK*-list, then *C* updates the *PK*-list with tuple $\left(ID_{B_{j}},⊥,pk_{B_{j}}^{{}^{\prime }}\right)$ and sets *x*_*B*_*j*__ = ⊥, $pk_{B_{j}}=pk_{B_{j}}^{{}^{\prime }})$.
- Secret key query: When *C* receives a secret key query on *ID*_*B*_*j*__, if *C* replaces public key of *ID*_*B*_*j*__, then *C* returns ⊥. Otherwise, there exists (*ID*_*B*_*j*__, *x*_*B*_*j*__, *pk*_*B*_*j*__) in the *PK*-list and returns *x*_*B*_*j*__ as answer.
- Unsigncrypt query: When receiving an unsigncrypt query under *ID*_*A*_, *ID*_*B*_*j*__ and ciphertext *σ*, if $ID_{B_{j}}\neq ID_{B_{i}}^{*}\left(i=1,2,\cdots ,n\right)$, *C* runs the formal unsigncrypt algorithm and return the answer. Otherwise, *C* goes through the *H*_4_-list with (*V*_*j*_, *h*_4_) to find a value such that *h*_4_ meets the VDBDH oracle returns 1 when queried on the tuple (*bP*, *c*^−1^ *P*, *U*_1_, *V*_*j*_). If such a tuple exists, return *h*_4_ and computer *r*_2_ = *φ*_*j*_ ⊕ *h*_4_, *K* = *KDF*(*r*_2_). Recover *M* = *DEM*.*Dec*(*K*, *C*), use *H*_5_ query to obtain *h*_*j*_, then check equation *e*(*P*_1_, *S*_*j*_) = *e*(*P*, *U*_2_ + *h*_*j*_*Q*_*A*_). If holds, return *M*. Otherwise, output ⊥.

Challenge: After the first stage, *A*_1_ outputs two plaintexts *M*_0_, *M*_1_ and *ID*_*A*_, *ID*_*B*_*j*__(*j* = 1, 2, ⋯, *n*) to *C*. If $ID_{B_{j}}\neq ID_{B_{i}}^{*}\left(i=1,2,\cdots ,n\right)$, then *C* fails and aborts. Otherwise, *C* randomly chooses $x^{*}\in Z_{q}^{*}$, $\varphi_{j}^{*}\in Z_{q}^{*}$, obtains $h_{j}^{*}$ from *H*_5_ query, sets $U_{1}^{*}=aP$, and computes $U_{2}^{*}=-h_{j}^{*}Q_{A}+x^{*}P_{1}$. Obtain $r_{2}^{*}=\phi_{j}^{*}⊕T$(where *T* is *C* candidate for the VDBDH obtained from *H*_4_ query), $K_{1}=KDF\left(r_{2}^{*}\right)$. Then, *C* randomly selects *K*_0_ ∈ *K*_*MHHSC*_ and *b* ∈ {0, 1} computes C* = *DEM*.*Enc*(*K*_*b*_, *M*_*b*_), $S_{j}^{*}=x^{*}P$. Finally, *C* provides the ciphertext $\sigma^{*}=\left(C^{*},\phi^{*}\leftarrow \left(U_{1}^{*},U_{2}^{*},S_{j}^{*},\phi_{j}^{*}\right)\right)$ to *A*_1_.

Phase 2. *A*_1_ request a second series of queries as before.

Guess: At the end of the simulation, *A*_1_ outputs a bit *b*′ for which the relation σ* = Signcrypt(*M*_*b*_, *sk*_*A*_, *ID*_*B*_*j*__) holds. If *b*′ = *b*, *C* outputs *T* = *e*(*U*_1_, *D*_*B*_*j*__) = *e*(*aP*, *bc*^−1^ *P*) = (*P*, *P*)^*abc*^−1^^ as a solution of the VDBDH problem.

Then, we assess probability. The probability to fail in signcryption queries is at most (*q*_*H*_4__ + *q*_*s*_)*q*_*s*_/2^*k*^, and the probability to fail in unsigncryption queries is at most *q*_*u*_/2^*k*^. Note that the probability for *C* to not to fail in first stage is (*q*_*H*_3__ − *q*_*ppk*_)/*q*_*H*_3__. Furthermore, with a probability exactly 1/(*q*_*H*_3__ − *q*_*ppk*_), *A*_1_ chooses to be challenged on $ID_{B_{i}}^{*}$. Thus, the advantage of *C* is $\frac{\epsilon -\left(q_{u}-q_{H_{4}}q_{s}-q_{s}^{2}\right)/2^{k-1}}{2q_{H_{3}}}$.

*Lemma 2. In the random oracle, if there is an IND-CCA2 adversary* *A*_2_ *has an advantage* *ϵ* *against MHHSC. Then an algorithm* *C* *that solves the DBDH problem with an advantage* $\frac{\epsilon -\left(q_{u}-q_{s}\right)/2^{k-1}}{2q_{H_{3}}}$.

Proof. We construct a simulator *C* uses *A*_2_ to decide whether *T* = *e*(*P*, *P*)^*abc*^ by providing a random instance (*P*, *aP*, *bP*, *cP*, *T*) as the DBDH problem. This proof considers the indistinguishability of *m*_*j*_.

Setup: At the beginning, *C* sets *P*_2_ = *s*_2_ *P* and proves the system parameters to the attacker *A*_2_. The target identity is $ID_{B_{i}}^{*}\left(i=1,2,\cdots ,n\right)$.

Phase 1. *A*_2_ requests a number of queries. *C* keeps the *H*_*i*_-list (*i* = 1, 2, 3, 4, 5) and *PK*-list, which are used to record answers to the corresponding *H*_*i*_ query and public key query.

- *H*_3_ query: Input an identity *ID*_*B*_*j*__. If $ID_{B_{j}}\neq ID_{B_{i}}^{*}\left(i=1,2,\cdots ,n\right)$, randomly choose $t_{j}\in Z_{q}^{*}$, calculates *Q*_*B*_*j*__ = *t*_*j*_ *P*. Otherwise, calculates *Q*_*B*_*j*__ = *bP* put (*ID*_*B*_*j*__, *t*_*j*_, *Q*_*B*_*j*__) in *H*_3_-list return *Q*_*B*_*j*__.
- *H*_*i*_(*i* = 0, 1, 2, 4, 5) query: Upon receiving an *H*_*i*_ query, if the corresponding query exists in the *H*_*i*_-list, then return it to *A*_2_. Otherwise, *C* randomly selects an integer as the query result and returns it to *A*_2_. Meanwhile, *C* places the query result into the *H*_*i*_-list.
- Public key query: Upon receiving a public key query on *ID*_*B*_*j*__. If $ID_{B_{j}}\neq ID_{B_{i}}^{*}\left(i=1,2,\cdots ,n\right)$, randomly selects $x_{B_{j}}\in Z_{q}^{*}$ computes *pk*_*B*_*j*__ = *x*_*B*_*j*__ *P* and updates the *PK*-list. If $ID_{B_{j}}=ID_{B_{i}}^{*}\left(i=1,2,\cdots ,n\right)$, set *pk*_*B*_*j*__ = *cP* update the *PK*-list with (*ID*_*B*_*j*__, ⊥, *cP*) and return *pk*_*B*_*j*__.
- Secret key query: When *C* receives a secret key query on *ID*_*B*_*j*__, if $ID_{B_{j}}=ID_{B_{i}}^{*}\left(i=1,2,\cdots ,n\right)$ returns ⊥. Otherwise, there exists (*ID*_*B*_*j*__, *x*_*B*_*j*__, *pk*_*B*_*j*__) in *PK*-list returns *x*_*B*_*j*__.
- Unsigncrypt query: When receiving an unsigncrypt query under *ID*_*A*_, *ID*_*B*_*j*__ and ciphertext *σ*, *C* can compute *V*_*j*_ = *e*(*U*_1_, *D*_*B*_*j*__), obtains *r*_2_ = *φ*_*j*_ ⊕ *H*_4_(*V*_*j*_), *K* = *KDF*(*r*_2_), recovers *M* = *DEM*.*Dec*(*K*, *C*). Then, if $ID_{B_{j}}=ID_{B_{i}}^{*}\left(i=1,2,\cdots ,n\right)$, *C* fails and stops(*C* cannot compute *R*_*j*_ for *sk*_*B*_*j*__ is only *ID*_*B*_*j*__ can compute). Otherwise, *ID*_*B*_*j*__ recovers its own message *m*_*j*_ = (*m*_*j*_ ⊕ *R*_*j*_) ⊕ *R*_*j*_. Submitting *H*_5_ query to obtain *h*_*j*_. Then, equation *e*(*P*_1_, *S*_*j*_) = *e*(*P*, *U*_2_ + *h*_*j*_ *Q*_*A*_) is checked. If holds, *m*_*j*_ is returned. Otherwise, output ⊥.

Challenge: After the first stage, *A*_2_ outputs two plaintexts *m*_0_, *m*_1_ and *ID*_*A*_, *ID*_*B*_*j*__(*j* = 1, 2, ⋯, *n*) to *C*, if $ID_{B_{j}}\neq ID_{B_{i}}^{*}\left(i=1,2,\cdots ,n\right)$, *C* fails and abort. Otherwise, *C* randomly chooses $x^{*}\in Z_{q}^{*}$, $\phi_{j}^{*}\in Z_{q}^{*}$, obtains $h_{j}^{*}$ from *H*_5_ query, sets $U_{1}^{*}=aP$, computes $U_{2}^{*}=-h_{j}^{*}Q_{A}+x^{*}P_{1}$, $V_{j}^{*}=e\left(U_{1},D_{B_{j}}\right)$. Gets $r_{2}^{*}=\phi_{j}^{*}⊕H_{4}\left(V_{j}^{*}\right)$, $K^{*}=KDF\left(r_{2}^{*}\right)$. computes $S_{j}^{*}=x^{*}P$, C* = *DEM*.*Enc*(K*, M*), where M* = *m*_*b*_ ⊕ *T*(*T* is *C* candidate for the DBDH). Finally, *C* provides the ciphertext $\sigma^{*}=\left(C^{*},\phi^{*}\leftarrow \left(U_{1}^{*},U_{2}^{*},S_{j}^{*},\phi_{j}^{*}\right)\right)$ to *A*_2_.

Phase 2. *A*_2_ then requests a second series of queries as before.

Guess: At the end of the simulation, *A*_2_ outputs a bit *b*′ for which believes the relation σ* = Signcrypt(M*, *sk*_*A*_, *ID*_*B*_*j*__) holds. If *b*′ = *b*, *C* outputs $T=e{\left(pk_{B_{j}},Q_{B_{j}}\right)}^{r_{1}}$ = *e*(*cP*, *bP*)^*a*^ = (*P*, *P*)^*abc*^ as a solution of DBDH problem.

Then, we assess probability. The probability to fail in signcryption queries is at most *q*_*s*_/2^*k*^, and the probability to fail in unsigncryption queries is at most *q*_*u*_/2^*k*^. Note that the probability for *C* to not to fail in first stage is (*q*_*H*_3__ − *q*_*sk*_)/*q*_*H*_3__. Furthermore, with a probability exactly 1/(*q*_*H*_3__ − *q*_*sk*_), *A*_2_ chooses to be challenged on $ID_{B_{i}}^{*}$. Thus, the advantage of *C* is $\frac{\epsilon -\left(q_{u}-q_{s}\right)/2^{k-1}}{2q_{H_{3}}}$.

### Unforgeability

*Theorem 2. In the random oracle model, if an EUF-CMA adversary* *F* *has the advantage* *ϵ* *against MHHSC, then exists an algorithm* *C* *that solves the VCBDH problem with the advantage* $\frac{\epsilon (1-\left(q_{H_{4}}+q_{s}\right)q_{s}/2^{k})}{q_{H_{1}}-q_{sk}}$.

Proof. We construct a simulator *C* that uses *F* to decide whether *e*(*P*, *P*)^*abd*^−1^^ by providing a random instance (*P*, *aP*, *bP*, *dP*, *d*^−1^) as the VCBDH problem.

Setup: At the beginning, *C* sets *P*_1_ = *dP* and provides the system parameters to the attacker *F*. The target identity is $ID_{A}^{*}$

Attack: *F* requests a number of queries. *C* keeps the *H*_*i*_-lists (*i* = 1, 2, 3, 4, 5) which are used to record answers to the corresponding *H*_*i*_ query.

- *H*_1_ query: Input an identity *ID*_*A*_. If $ID_{A}\neq ID_{A}^{*}$, $t\in Z_{q}^{*}$ is randomly selected, *Q*_*A*_ = *tP* is calculated. Otherwise, calculate *Q*_*A*_ = *bP* place (*ID*_*A*_, *t*, *Q*_*A*_) into the *H*_1_-list, and return *Q*_*A*_.
- *H*_*i*_(*i* = 0, 2, 3, 4, 5) query: Upon receiving a *H*_*i*_ query, if the corresponding query exists in the *H*_*i*_-list, return it to *A*_2_. Otherwise, *C* randomly selects an integer as the query result and returns it to *A*_2_. Meanwhile, *C* places the query result into the *H*_*i*_-list.
- Private key query: When *C* receives a partial private key query on *ID*_*A*_, if $ID_{A}\neq ID_{A}^{*}$ retrieves the corresponding (*ID*_*A*_, *t*, *Q*_*A*_) from the *H*_1_-list and sets *sk*_*A*_ = *td*^−1^ *P*, return *sk*_*A*_. Otherwise, *C* aborts.
- Signcrypt query: When receiving a signcrypt query under *ID*_*A*_, ${\left\{ID_{B_{i}}\right\}}_{i=1}^{n}$ and *n* messages *m*_*i*_(*i* = 1, 2, ⋯, *n*). If $ID_{A}\neq ID_{A}^{*}$, the formal signcrypt algorithm runs and returns ciphertext *σ*. Otherwise, *C* randomly selects $x,r_{2}\in Z_{q}^{*}$, computes *U*_1_ = *xP*_2_, *V*_*i*_ = *e*(*U*_1_, *D*_*B*_*i*__), *R*_*i*_ = *e*(*U*_1_, *sk*_*B*_*i*__), *φ*_*i*_ = *r*_2_ ⊕ *H*_4_(*V*_*i*_) and let *φ* = (*φ*_1_, *φ*_2_, ⋯, *φ*_*n*_). Compute *C* = *DEM*.*Enc*(*K*, *M*) where *K* = *KDF*(*r*_2_) and *M* = (*m*_1_ ⊕ *R*_1_‖*m*_2_ ⊕ *R*_2_‖⋯‖*m*_*n*_ ⊕ *R*_*n*_). Obtain *h*_*i*_ from the *H*_5_ query, compute *U*_2_ = −*h*_*i*_*Q*_*A*_ + *xP*_1_, *S*_*i*_ = *xP*, and return ciphertext *σ* = (*C*, *ϕ* ← (*U*_1_, *U*_2_, *S*, *φ*)).

Equation *e*(*P*_1_, *S*_*i*_) = *e*(*P*, *U*_2_ + *h*_*i*_*Q*_*A*_) holds.

Forge: Finally, *F* outputs σ* and *ID*_*A*_, *ID*_*B*_*i*__ to *C*. If $ID_{A}\neq ID_{A}^{*}$, *C* fails and aborts. Otherwise, by forking lemma [25], *C* selects a different hash function *h*_*i*_ and interacts with *F* with the same random tape, then the adversary *F* can provide a different forger *σ*′*. We know that σ* and *σ*′* should satisfy the equation $e\left(P_{1},S_{i}^{*}\right)=e\left(P,U_{2}+h_{i}^{*}Q_{A}\right)$ and $e\left(P_{1},S_{i}^{{}^{\prime }*}\right)=e\left(P,U_{2}+h_{i}^{{}^{\prime }*}Q_{A}\right)$. $bd^{-1}P=\frac{S_{i}^{*}-S_{i}^{{}^{\prime }*}}{h_{i}^{*}-h_{i}^{{}^{\prime }*}}$, is obtained, then *C* derives the value of *e*(*P*, *P*)^*abd*^−1^^ as *e*(*aP*, *bd*^−1^ *P*). Hence, *C* successfully solves the VCBDH problem.

The probability of failing in signcryption queries is at most (*q*_*H*_4__ + *q*_*s*_)*q*_*s*_/2^*k*^. With a probability of exactly 1/(*q*_*H*_1__ − *q*_*sk*_), *F* chooses to be challenged on $ID_{A}^{*}$. Then, the advantage of *C* is $\frac{\epsilon (1-\left(q_{H_{4}}+q_{s}\right)q_{s}/2^{k})}{q_{H_{1}}-q_{sk}}$.

## Performance evaluation

### Functionality comparison

To our knowledge, no hybrid signcryption schemes have achieved heterogeneity. Therefore, we compare our scheme with existing heterogeneous signcryption schemes [6] [8] in terms of supporting multi-message, multi-recipient, identity privacy-preservation, heterogeneous system, and different master keys. Table 1 illustrates that our scheme has many excellent features. First, the scheme takes advantages of pseudo-identity to ensure the anonymity of senders and receivers. Second, the scheme supports heterogeneous systems with different master keys. Our scheme has more advantages from the functionality and system setup perspective.

**Table 1:** Functionality comparison.

Comparison items	Our scheme	[6]	[8]
Heterogeneous system	Yes	Yes	Yes
Master key	Yes	No	Yes
Multi-message	Yes	No	No
Multi-recipient	Yes	No	Yes
Privacy-preservation	Yes	No	Yes

Then, we compare the computational costs of scheme [8] with that of our scheme. In scheme [8], numerous additions and multiplications must be executed to computing *p*_*i*_(*x*) and *F*_*i*_. If the steps of computing *p*_*i*_(*x*) and *F*_*i*_ are not considered, Table 2 shows that scheme [8] still requires 7P, 6M and 3E, thus indicating that it is less efficient than our scheme. Here P, M, and E denote pairing, multiplication, and exponentiation operations, respectively.

**Table 2:** Computational cost.

Scheme	Signcryption	Unsigncryption
[8]	2P + 3M + 1E	5P + 3M + 2E
Ours	2P + 1M + 2E	4P + 1M

P: pairing operation; M: multiplication operation; E: exponentiation operation.

### Computational overhead comparison

To provide numerical results, we implement IBC-CLC MHHSC to measure the performance of signcryption and unsigncryption operations. Our implementation is written in C using the Pairing-Based Cryptography Library (Libpbc) [26]. For the computations, we use the curve groups that are implemented in the Libpbc library. The computations are run on a PC with 3.10 GHz CPU frequency, 4 GB of RAM, and Linux operating system. In the experiment, we used elliptical curves with a base field size of 512 bits and an embedding degree of 2. The security levels are selects as |*p*| = 512.

**Fig 1:**
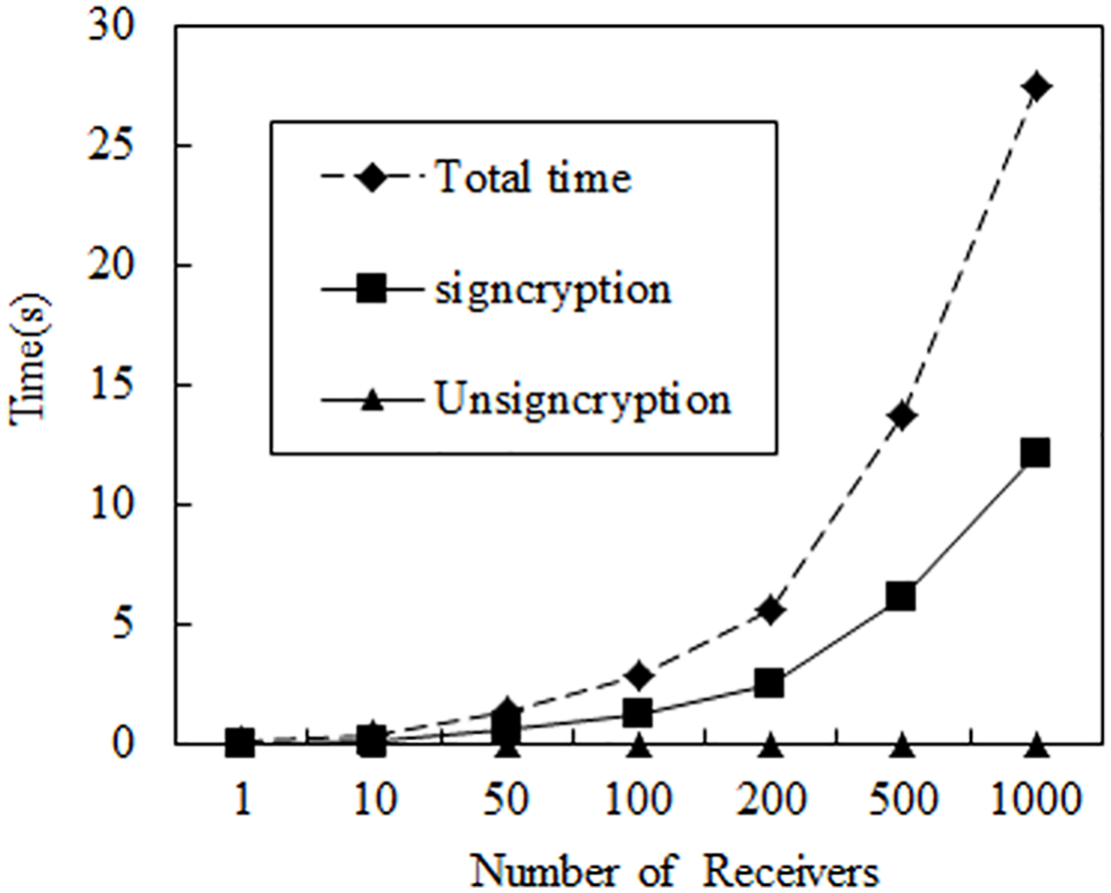
Operation time(s). (Unsigncryption time near the bottom of the coordinate axis).

The performing consequence of our scheme is provided in Fig 1. Including total operation, signcryption, and unsigncryption operation time of our scheme when the number of the receiver is set as *n* = 1, 10, 50, 100, 200, 500, 1000. From the figure, we can indicate that signcryption time increases with the number of recipients. However, when unsigncryption, each receiver only operates on its own message, the unsigncryption operation time is not related to the increase of the receiver. So compared with the signcryption and total operation time of the receiver for 1000, the unsigncryption operation time is 0.018, near the bottom of the axis. Therefore, we can see that our scheme can achieve more efficient communication between two systems, which have greater difference in computing power. Users in IBC can handle big data, while users in CLC only need deal with a few data, such as infrastructure-to-vehicle (I2V) communication in vehicular ad hoc networks (VANETs). Trusted authorities or road side units can be the users in IBC system, which have much more capability, and hundreds of on board units can be the users in CLC system, which ability is limited.

## Conclusion

We propose a novel conditional privacy-preserving heterogeneous hybrid signcryption scheme for IBC to CLC (MHHSC), which allows to send multi-message to multi-receiver. The proposed scheme selects different master secret keys in different systems and maps a distinct pseudo-identity for each user, only the trusted authority could trace the real identity for any disputed event when necessary, which ensures conditional privacy preservation for all users in heterogeneous systems. It is definitely more practical for actual applications, such as VANETs. Moreover, we provide the formal definition and security models for the heterogeneous hybrid signcryption scheme. Proof shows that our scheme is indistinguishability against adaptive chosen ciphertext attacks and existential unforgeability against adaptive chosen message attacks, which is satisfied confidentiality and unforgeability in the random oracle model. Owing to today’s diverse and complex network system and application environment, our follow-up work could be propose a bidirectional heterogeneous signcryption scheme between IBC and CLC for multi-party user.
